# The MoSGrid - e-science gateway: molecular simulations in a distributed computing environment

**DOI:** 10.1186/1758-2946-5-S1-O3

**Published:** 2013-03-22

**Authors:** Lars Packschies, Georg Birkenheuer, Dirk Blunk, Sebastian Breuers, André Brinkmann, Ines dos Santos Vieira, Gregor Fels, Sandra Gesing, Richard Grunzke, Sonja Herres-Pawlis, Oliver Kohlbacher, Jens Krüger, Martin Kruse, Ulrich Lang, Ralph Müller-Pfefferkorn, Patrick Schäfer, Tobias Schlemmer, Thomas Steinke, Klaus-Dieter Warzecha, Andreas Zink

**Affiliations:** 1 Universität zu Köln, 50923 Köln, Germany; 2 Universität Paderborn, 33098 Paderborn, Germany; 3 Technische Universität Dortmund, 44221 Dortmund, Germany; 4 Ludwig-Maximilians-Universität München, 81377 München, Germany; 5 Technische Universität Dresden, 01069 Dresden, Germany; 6 Eberhard Karls Universität Tübingen, 72074 Tübingen, Germany; 7 Zuse Institut Berlin, 14195 Berlin, Germany

## 

Modern tools for computational chemistry allow the calculation of a wide range of properties of all sorts of molecules applying various levels of theory. But to perform convincing and significant calculations with these tools not only requires insight into the scientific theory itself, but also knowledge and experience on how to operate the simulation tools.

In addition to the general challenge of gaining access to a powerful computing environment, very often a high level of technical competence is necessary to set up and run calculations efficiently. These prerequisites often hamper scientists to routinely use computational tools to support or confirm their perceptions.

To overcome some of these problems, the MoSGrid consortium develops an open source e-science portal for grid based environments with respect to computational chemistry. At present residing in the German Grid Initiative (D-Grid), MoSGrid enables users to set up, run and evaluate calculations using tools from the domains of Quantum Chemistry, Molecular Dynamics and Docking [1].

This talk underlines the basic motivation, layout, development, properties and available tools of MoSGrid as well as the procedure of gaining access to the grid environment.
